# “A good death but there was all this tension around”- perspectives of residential managers on the experience of delivering end of life care for people living with dementia

**DOI:** 10.1186/s12877-021-02241-7

**Published:** 2021-05-12

**Authors:** Jessica A. L. Borbasi, Allison Tong, Alison Ritchie, Christopher J. Poulos, Josephine M. Clayton

**Affiliations:** 1 Centre for Learning & Research in Palliative Care, HammondCare, Greenwich Hospital, Sydney, Australia; 2 The University of Sydney, School of Public Health, Faculty of Medicine and Health Sydney, Sydney, NSW Australia; 3 Centre for Positive Ageing, HammondCare, Hammondville, Sydney, Australia; 4 University of New South Wales, Sydney, Australia; 5 The University of Sydney, Northern Clinical School, Faculty of Medicine and Health, Sydney, Australia

## Abstract

**Background:**

End of life care for residents with advanced dementia in the aged care setting is complex. There is prolonged and progressive cognitive decline, uncertain disease trajectory, significant symptom burden and infrequent access to specialist palliative care. Residential aged care managers offer a unique perspective in understanding the experience of providing end of life care for residents with advanced dementia. They bring insight from the coalface to the broader policy context. The aim of this study was to describe the experience and perspectives of residential aged care managers on providing end of life care for residents living with dementia.

**Methods:**

Focus groups and semi-structured interviews were conducted with residential or care managers from various care homes from one dementia specific aged care organisation in Australia. A comprehensive sampling strategy was used in participating care homes. Transcripts were analysed using thematic analysis.

**Results:**

20 residential or care managers from 11 aged care homes in two states of Australia participated in two focus groups (total 16 participants) or individual interviews (4 participants). Six themes were identified: laying the ground work to establish what families understand about dementia, playing the peacemaker in the face of unrealistic family demands and expectations, chipping away at denial and cultivating a path towards acceptance of death, recruiting general practitioners as allies, supporting and strengthening the front line, and dedication to optimal care is relentless but rewarding.

**Conclusion:**

Aged care manager participants described provision of end of life dementia care as a rewarding but sometimes fraught experience requiring persistent personalisation of care and communication to enable family acceptance of the resident’s terminal condition. The findings suggest that continuous front line aged care staff skill development, iterative family discussions, and partnership building between aged care staff and general practitioners, are all required to promote optimal end of life dementia care in residential aged care settings.

## Background

Dementia has an increasing prevalence affecting approximately 47 million people worldwide, and is for many eventually a terminal condition [1]. Progressive functional decline often precipitates the need for full time care in residential aged care homes [2, 3]. In Australia, the majority of people in aged care have dementia [2]. Despite having palliative care needs comparable to those dying of cancer, people with advanced dementia often receive futile and even painful interventions at the end of life and are unlikely to receive specialist palliative care [3–6]. There is a great need for generalist palliative care for people living with advanced dementia, particularly in residential aged care [7, 8].

The challenges to providing palliative dementia care include prolonged and progressive cognitive decline that limits the persons’ capacity to be involved in decisions about their own care, and the unpredictable trajectory of dementia leading to uncertainty about the appropriate timing of a palliative approach [9]. Other barriers include lack of staff training in dementia and palliative care, inadequate advance care planning; time constraints, poor interdisciplinary communication and a misperception, from families and professionals alike, that dementia, on its own, is not a life limiting illness [9–12]. Professionals in the aged care setting, including some managers, have also identified a general lack of knowledge and skill relating to the palliation of people with advanced dementia [13–15].

Few studies have examined the challenges of caring for people with advanced dementia at the end of life from the perspectives of residential aged care managers. Residential managers afford a distinct viewpoint in appreciating the complexities of dementia care from the coalface to the broader policy context. Support from residential managers is also necessary for the development and implementation of palliative care in the residential aged care settings [16]. This study aims to describe the experiences and challenges faced by managers when delivering end of life care to residents with advanced dementia.

## Methods

We used the consolidated criteria for reporting qualitative health research to report this study [17].

### Participant selection and practice setting

In Australia residential aged care is for older Australians who are no longer able to live independently and is government funded. Residential care providers differ in the nature of the personal, nursing and general health care services available in their facility.

Participants were eligible if they were a residential manager or care manager at HammondCare, a non-profit residential aged care organisation, which operates a dementia specific, and mostly a clustered cottage model approach to residential aged care [18]. The organisations’ care homes ranged from 40 to 146 beds, with each care home typically organised into 10–18 bed domestic clusters. The residential manager roles in the organisation range from operation managers who have oversight of several care homes to service managers, clinical care managers, and assistant managers in individual care homes. Using a comprehensive sampling approach, we informed all potentially eligible participants from across the organisation about the opportunity to participate in a focus group or individual interview. Potential participants were informed about the study via email invitation and presentations and flyers at staff meetings. All participants were fully informed and provided written consent to take part in the study including the subsequent publication of de-identified findings. The study was approved by the University of Sydney Human Research Ethics Committee (2018/744).

### Data collection

The semi-structured interview and focus group guide (appendix I) were informed by a literature review and discussion among the research team. The interview guide was piloted with staff not currently providing clinical care in the organisation prior to its use in the interviews. The two focus groups were held in December 2018 and January 2019, each in different geographic networks within the organisation to enable a range of managers from different care homes to participate. Interviews were offered to potential participants who expressed interest in taking part in the study but who were unable to attend one of the focus groups. Two researchers not known to the participants conducted the focus groups (including A.T. & C.K.). A palliative care specialist doctor in training (J.B.), who was also not known to the participants nor involved in providing clinical care to the study sites, conducted the telephone interviews from December 2018 and May 2019. Participants were informed that the purpose of the focus groups and interviews was to enable quality improvement. No one was present in the interviews or focus groups aside from the participants and researchers. Participant recruitment ceased when data saturation was reached. All research personnel had completed training in qualitative research methods. Interviews and focus groups were digitally audio-recorded and transcribed. Transcripts were de-identified and assigned codes by a research assistant (A.R.) prior to being sent to the wider research team for analysis. The researchers also maintained field notes to capture their perceptions during data collection.

### Data analysis

Drawing on grounded theory and thematic analysis, J.B. inductively identified and recorded concepts in the transcripts relating to participants’ experiences and perspectives regarding provision of end of life care for residents living with dementia [19]. Codes were generated from the entire data set. Similar codes were grouped as potential themes and reviewed until finally an overarching thematic map was generated. To enhance the analytical framework, members of the researcher team independently familiarised themselves with the data and then discussing themes and meaning together.

## Results

Of the approximately 35 potential participants from all 14 of HammondCare’s care homes in two states of Australia (New South Wales and Victoria) who were invited to participate, 20 managers from 11 residential aged care homes in these two states participated in two face-to-face focus groups (8 participants each) and four telephone interviews. The main reason for non-participation, where provided, was time constraints. Between one and two managers from each of the 11 care homes participated, plus 4 managers with operational roles across several care homes. The majority of participants were female, and participants were aged from 27 to 62 years (Table 1). Years of experience in aged care ranged from 2 to 26 years. Most (*n* = 16) had formal training in healthcare, most commonly in nursing (*n* = 14). Half had received palliative care training “on the job” and most participants received dementia care training via short courses. Semi-structured interviews lasted on average 42 min (range 33–52 min). Focus groups lasted on average 116 min (range 116–117 min).

**Table 1:** Participant Characteristics.

Characteristics	Participants (***n*** = 20)
Mean age, *y*	40
Female, *n (%)*	12 (60)
Country of birth, *n (%)*	
Australia	17 (85)
Level of education, *n (%)*	
Undergraduate	11 (55)
Professional certificate	4 (20)
Postgraduate	2 (10)
Formal training in health care*, *n (%)*	16 (80)
Formal training in aged care, *n (%)*	16 (80)
Mean years of experience in aged care, *y*	12
Training in palliative care, *n (%)*	
On the job training	10 (50)
vShort course	9 (45)
Training in dementia care, *n (%)*	
On the job training	5 (25)
Short course	12 (60)
Course with qualification	3 (15)

*14 registered nurses, 1 psychologist and 1 diversional therapist

**Fig. 1:**
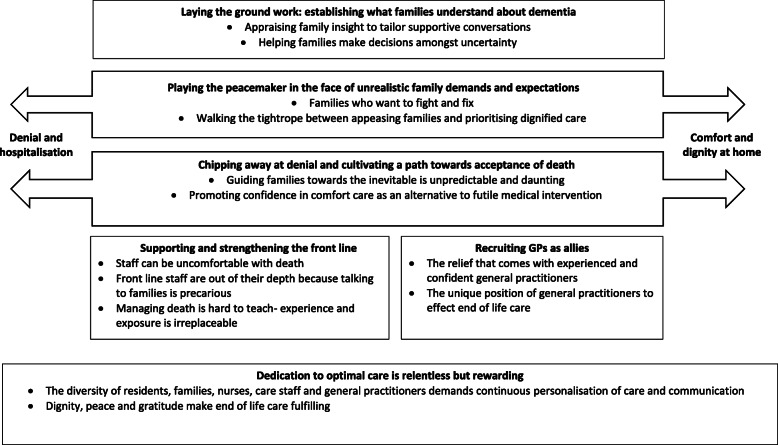
Thematic representation.

Six major themes were identified: 1) laying the ground work; establishing what families understand about dementia, 2) playing the peacemaker in the face of unrealistic family demands and expectations, 3) chipping away at denial and cultivating a path towards acceptance of death, 4) recruiting GPs as allies, 5) supporting and strengthening the front line, and 6) dedication to optimal care is relentless but rewarding. The themes and subthemes are described in the following section, additional illustrative quotations by participant number and thematic representation are provided in Table 2 and Fig. 1, respectively.

**Table 2:** Selected illustrated quotations by participant number.

Themes and subthemes	Illustrative quotations
***Laying the ground work: establishing what families understand about dementia***	
*Appraising family insight to tailor supportive conversations*	“Dementia is a terminal illness, but lots of people out there don’t know that. If people understood better, I think there would be a roll-on effect … and the ability to talk about it would become easier because people know that it’s coming.” (56, interview) “Often the person [family] will say I don’t want to talk about that now, I will talk to the doctor in the hospital about that.” (14, focus group 2).
*Helping families make decisions amongst uncertainty*	“Sometimes the family does not have any idea about what palliative care is, we have to educate them from the beginning” (9, focus group 1) “I don’t think we have a consistent approach to advance care planning. We don’t even have a form that’s a consensus across the company.” (14, focus group 2)
***Playing the peacemaker in the face of unrealistic family demands and expectations***	
*Families who want to fight and fix*	“The daughter wants full active treatment, and wants them force fed, and wants full CPR [cardiopulmonary resuscitation] on a 90-year-old man who weighs 48 kg, and is not really aware of his surroundings because he’s got advanced dementia. That’s a huge ethical dilemma for me because I don’t think we should be intervening” (6, focus group 1) “They [a family] think if their loved one is not eating or drinking towards the end of life that we’re starving them to death. We have to explain to them that at this stage of life the person doesn’t actually feel like anything to eat or drink.” (10, interview)
*Walking the tightrope between appeasing families and prioritising dignified care*	“They’re unconscious, in a coma, and near death. Then you’ll have a friend or a family member walk in, who hasn’t been there throughout that journey, and say, oh you’re giving them morphine, that’s why they’re like that” (4, focus group 1). “Everything went completely pear-shaped. The family were sure that because we’d given the most miniscule of comfort medications to this lady, who really needed them, they felt we should put euthanasia on the death certificate. That was heart breaking - we spent hours talking to them [the family] but we still failed. There was this balance between distressing them and distressing her” (14, focus group 2).
***Chipping away at denial and cultivating a path towards acceptance of death***	
*Guiding families towards the inevitable is an unpredictable and daunting process*	“It’s about relationship-building and making the family comfortable enough to be able to release their loved ones into our care, to safely send them on their journey” (17, focus group 2). “The sheer unknown can be daunting at times when you’re not quite sure how family or a GP or a resident or a staff member are going to respond [to end of life discussions]” (18, focus group 2).
*Promoting confidence in comfort care as an alternative to futile medical intervention*	“I talk about all the things that we do to stay out of hospital and I leave it at that. Sometimes you’ll get pushback [from families] and that’s something you need to slowly chip away “(18, focus group 2). “So all those complaints, those emails, the meetings, all that stuff is irrelevant. To see him [the son] acknowledge that this is her home, and that we need to care for her here -- was really quite a nice experience” (6, focus group 1).
***Recruiting GPs as allies***	
*The relief that comes with experienced and confident general practitioners*	“Then you get an after-hours doctor that doesn’t turn up, or doesn’t want to come, or refuses to assist you because they don’t want to get involved, because it’s end of life” (6, focus group 1). “We’ve got some amazing GPs, and half your battle is working with the GPs” (16, focus group 2).
*The unique position of general practitioners to effect end of life care*	“They (GPs) want to help people, they want to send people for tests, they want to make people better. Palliative care is not a concept that sits comfortably for them” (6, focus group 1). “I had a GP who wasn’t sure what to do, so I gave him the palliative book. He read that, was happy, took a copy home for himself and prescribed accordingly” (18, focus group 2).
**Supporting and strengthening the front line**	
*Staff can be uncomfortable with death*	“It depends on your staff and their education, how comfortable they are at recognising and delivering end of life care, as well as communicating it. Because if you’re not comfortable giving it, then you’re not comfortable communicating it to families and friends “(16, focus group 2).
*Front line staff are out of their depth because talking to families is precarious*	“Care staff indicate they want to do a course on difficult conversations. I’m still yet to understand what that means, but essentially what they’re saying is they’re not comfortable and they believe that there’s a course or some sort of definitive way to manage all these scenarios.” (18, focus group 2). “They [care staff] don’t seem to have the skill to be malleable with their communication style ... they don’t pick up cues from the families” (10, interview).
*Managing death is hard-to-teach; experience and exposure is irreplaceable*	“Theory is one thing, but actually putting it into clinical practice and being exposed to it teaches you ten times what the textbook can” (10, interview). “The more times you experience end of life care adds to your abilities and each death is so different, you take something away from each person” (5, interview).
**Dedication to optimal care is relentless but rewarding**	
*The diversity of residents, families, nurses, care staff and general practitioners demands continuous personalisation of care and communication*	“It’s about knowing the individual resident and tailoring care to them, knowing your staff individually, and their strengths and weaknesses, and competency with palliative care. Knowing the families individually and where they’re up to in the journey. There’s a very difficult intersection between all of these things, that we just try to manage.” (3, focus group 1)
*Dignity, peace and gratitude make end of life care fulfilling*	“Supporting someone to die well is just the most amazing experience. It’s scary the first time, but I think it’s the most amazing experience because it’s the ultimate in caring and supporting.” (55, interview) “I think most of the time the vast majority of people that we support through palliative care do end up having good deaths and it’s not a traumatic experience for anyone.” (56, interview)

### Laying the ground work; establishing what families understand about dementia

#### Appraising family insight to tailor supportive conversations

Participants believed that time and expertise was needed to carefully ascertain a family’s knowledge of dementia and their future expectations. One participant said it was “a big signal if the family hope the person will get better and improve” as this will inevitably require “ongoing conversations” (14, focus group). However, participants noted that for some families, contemplating deterioration and death was “just not something you can discuss with them” (5, interview).

#### Helping families make decisions amongst uncertainty

Despite a diagnosis of advanced dementia, participants felt that many families were unaware of their loved ones wishes for medical intervention and end of life care. One participant stated that they have “never had anyone come through with a family certain of what their loved one wants at the end of life” (01, focus group). However, another participant remarked that it was “so hard for the families, because they’re trying to decide for someone they’re grieving over” (11, focus group).

### Playing the peacemaker in the face of unrealistic family demands and expectations

#### Families who want to fight and fix

Participants noted that some families did not understand the progressive nature of dementia and seemed unwilling to contemplate the likely future declining health of their relative. Participants believed this contributed to unrealistic expectations, such as families requesting intensive medical interventions like cardiopulmonary resuscitation. These interventions were perceived by participants to be counterproductive to good end of life care. In these situations participants described “a huge ethical dilemma” as they felt it was not appropriate to “be intervening” (006, focus group). A participant described a family who asked “what can we do? How can we fix this? and we [the managers] have to constantly say to them it’s not fixable. We can do this [palliate] but in the end … I don’t know whether it’s guilt but - it’s a fight to the bitter end” (14, focus group).

#### Walking the tightrope between appeasing families and prioritising dignified care

Participants felt they sometimes had to balance the family’s wishes against their own priorities to provide comfort care – it was “a battle because the family are so overwrought with ‘I don’t want to be responsible for my loved one’s death. So, I’m going to do everything I can to prevent my loved one from dying’” (14, focus group). The participants experienced tension and moral distress in advocating for residents’ rights for comfort care, in situations where families were seen to be refusing medications to alleviate the residents’ symptoms because, they were “not ready to let their relative die” (006, focus group).

### Chipping away at denial and cultivating a path towards acceptance of death

#### Guiding families towards the inevitable is an unpredictable and daunting process

Participants described trying to educate families about the progressive nature of dementia as “the more you can give people a picture of what it might be like [end of life], the better the experience will be for everyone. The caveat is that the family has got to be ready to hear that” (55, interview). As a residents’ health deteriorated participants responded to family concerns, distress and questions. Some participants found this process to be “daunting” and unpredictable, with a continuing need to renegotiate family expectations. If a resident started “to lose copious amounts of weight because of their dementia” participants intervened to set “expectations, of what we can control and what we can’t control and what’s inevitably going to happen” to prevent families being distressed about weight loss (10, interview).

#### Promoting confidence in comfort care as an alternative to futile medical intervention

Participants engaged in proactive, complex, reiterative discussions with families to instil confidence in palliative care. One participant described how “somebody was surprised that their loved one could stay here [for end of life care] and be with us. They thought they had to go to hospital.” (18, focus group). Participants believed that occasionally hospital transfer was an easier option. One participant remarked that “some families feel that the medical model is what end of life is, that people should be going to hospital to die” (18, focus group).

### Recruiting GPs as allies

#### The relief that comes with experienced and confident general practitioners

Participants looked to share the responsibility of end of life care with general practitioners (GPs) and hoped GPs would work with families to facilitate acceptance of palliative care. One participant described how “the GP, if they have any courage, will speak up to the family and advocate” (3, focus group). However, participants thought that only GPs who were experienced and confident in providing palliative care were able to do this. Participants reported challenges with accessing GPs who were willing to provide palliative care, particularly when urgently required out of hours. Other participants appreciated GPs who were “open to suggestions and responsive and who listen to our feedback” (5, interview) and they felt this made an enormous difference to being able to provide good end of life care.

#### The unique position of general practitioners to effect end of life care

Participants felt that GP support was instrumental in influencing family acceptance of a palliative approach and that GPs have an essential role in prescribing necessary medications and de-prescribing inappropriate medications. Participants strategized ways to partner with GPs and “get them on the journey with us” (12, focus group). Some participants observed that “there is almost a palpable fear from GPs” to be involved in palliative care (1, focus group), which compounds the difficulty in providing optimal end of life care.

### Supporting and strengthening the front line

#### Staff can be uncomfortable with death

Participants also highlighted the crucial role of front-line staff within the care home, including care workers, nurses, pastoral care staff and volunteers, to ensuring good end of life care and liaising with families. However, they felt that some front-line staff in particular nurses and care workers, were inexperienced and uncomfortable providing end of life care and this could jeopardise comfort care and relationships with families. One participant said how they have “come across some nurses who are afraid to give morphine, [because] they still think that ‘oh, if I give it and it’s the last dose I’ve killed them’ (10, interview).”

#### Front line staff are out of their depth because talking to families is precarious

Participants stated that careful and adaptable communication from care staff is needed to build rapport with families and facilitate their acceptance of palliative care. Miscommunication between care staff and families could lead to families being upset or seeking hospital care. The participants strived to have consistent and competent staff available to communicate with families about a residents’ care. However, participants thought that front line staff often lacked the communication skills required to undertake precarious and important conversations with families, despite them interacting with families on a daily basis. One participant described how for “a lot of our registered nurses English is a second language and so it’s difficult for them to articulate what they need to say, and also difficult for the families to understand them.” (10, interview).

#### Managing death is hard-to-teach; experience and exposure is irreplaceable

Participants felt that end of life care experience, both personal and professional, was far more valuable for staff within the care home than theoretical knowledge. One participant stated that he asked his staff “Have you ever seen a dead body? Have you ever had anyone in your life, a loved one or friend, relative, that’s passed away? Because half of them never have.” (6, focus group).

### Dedication to optimal care is relentless but rewarding

#### The diversity of residents, families, nurses, care staff and general practitioners demands continuous personalisation of care and communication

Participants described their role as all-encompassing to oversee provision of end of life care for residents. They were required to; understand the idiosyncrasies of residents and their families, build rapport and elicit family expectations early and know which staff are capable of communicating effectively with families and providing end of life care. Participants also need to know their residents’ GPs and whether they were “on the journey” with a comfort care approach or have the potential to jeopardise the relationship the managers and front line staff have cultivated with families. The managers stated that the stakes are high as family responses to discussions about end of life care were unpredictable and could range from gratitude to distress and requests for hospitalisation.

#### Dignity, peace and gratitude make end of life care fulfilling

Despite all the tension and challenges they experienced, participants reflected positively on how many families come to appreciate the care provided for residents as they approach the end of life and recognise the well-meaning actions of all the staff. One described how “for residents who may not let you brush their teeth or comb their hair, or dress them … we often get a lot of reward from being able to do that [at the end of life] because you feel like you’re restoring that person’s dignity” (1, focus group). Participants reported that families and staff also come together in their grief over a resident’s death. Overall participants found caring for residents with advanced dementia at the end of life to be a very rewarding and enriching experience.

## Discussion

Managers of residential aged care homes in this study described their role as overseeing and navigating a complex intersection between residents with advanced dementia, staff, GPs and families. Managers perceived that families of residents varied considerably in their understanding of dementia and their preparedness to participate in end of life discussions and decision making. Managers described how some families wanted to “fight and fix” the dementia and these families were perceived to seek medical intervention and hospital care. Managers tried to promote acceptance of death and hospital avoidance by iterative conversations chipping away at any misconceptions or denial, but sometimes found themselves balancing family wishes for life prolongation with their own desire to provide comfort directed care. Managers highlighted the critical role of GPs and front-line staff in communicating with families and providing end of life care for residents. However, the ability and willingness of GPs, nurses and care staff to provide this care was seen to be undependable. Ultimately the managers experienced the navigation of residents, families, care workers, nurses and GPs, who may all have different reactions to end of life dementia care, to be a relentless but rewarding vocation.

Despite their complex role, many of the managers did not have formal training in palliative or dementia care, although most had a health background in nursing. However, they were a reasonably experienced group possibly reflecting their own assertions that experience in end of life care is more important than textbook knowledge. Much of this expertise was used to navigate family dynamics.

In this study, managers believed that families were often unaware of the terminal nature of dementia and the care wishes of their loved ones, with advance care planning having rarely been completed prior to admission to the care home. Previous studies have similarly found that family members can pose a barrier to palliative care, particularly when they lack knowledge and understanding about the natural progression of dementia or are reluctant to discuss end of life care [8, 20–22]. Other research has highlighted how family members need more information from staff about dementia and palliative care to make decisions [20].

Managers perceived that misinformation regarding dementia and a reluctance to participate in advance care planning meant that families were more likely to request “futile” medical intervention. Uninformed families have previously been perceived to seek medical intervention, even in contradiction to their loved ones wishes described in an advance care directive [10]. Ultimately this results in decision making conflict and is known to be distressing for staff [10]. Conflict between residential staff and families has been highlighted in the literature as an ongoing challenge in aged care [23, 24]. In this study managers also perceived conflict within themselves as they felt morally compromised trying to promote comfort care for the resident whilst supporting families who they observed to have unrealistic goals of care.

General practitioners who could facilitate challenging conversations and prepare families for a resident’s death were valued by managers. Managers also relied on GPs to prescribe appropriate medications for comfort care. GPs were seen as essential to ensuring quality end of life care and for these reasons, managers endeavoured to collaborate with them. Previous research has also highlighted the importance of GPs for end of life care in aged care homes [8, 23]. However, in this study managers perceived great variability in the willingness, aptitude and availability of GPs which, is not dissimilar to experiences in the UK [23].

It is well established that individualised care is the foundation of dementia care and front-line, person centred care contributes to the rewarding nature of this work [25, 26]. Managers in this study highlighted the critical role of front-line staff, including nurses, care workers, pastoral care staff and volunteers within the care home, in providing person centred palliative care. However, managers thought front-line staff varied in their confidence, possibly due to a lack of experience, in delivering end of life medications and lacked the communication skills required to navigate diverse end of life discussions with families. Previous studies also revealed a reluctance of staff to participate in end of life care decision making and the importance of family and staff being in agreement regarding the goals of care [27–31].

### Limitations

The participants in this study only included managers from one organisation with a clustered cottage model approach to residential dementia care. Thus, the transferability of the findings beyond the setting may be uncertain. Due to time and funding reasons the transcripts were not returned to participants for comment or correction.

## Conclusion

Managers in residential aged care caring for residents dying with advanced dementia are willing to provide palliative care and find great reward in doing so, however, they feel hampered by challenging family requests, and uncertainty in navigating care with general practitioners and care staff. The findings suggest that continuous front line aged care staff skill development, reiterative family discussions to enable family acceptance of the resident’s terminal condition, and partnership building between aged care staff and general practitioners, are all required to promote optimal end of life dementia care. Residential aged care managers are in a unique position to effect change across organisations by influencing practice and training of front-line staff, contributing to policy and coordinating stakeholder relationships.

## Data Availability

Not applicable

## Abbreviations

GP: general practitioner

## References

[1] World Health Organisation. The Epidemiology and Impact of Dementia; Current state and future trends [Internet]. [cited 2020 Jul 17]. Available from: https://www.who.int/mental_health/neurology/dementia/dementia_thematicbrief_epidemiology.pdf

[2] Australian Institue of Health and Welfare. Dementia in Australia [internet]. 2012. Available from: https://www.aihw.gov.au/getmedia/199796bc-34bf-4c49-a046-7e83c24968f1/13995.pdf.aspx?inline=true. [cited 2020 Jul 17]

[3] Mccarty CE, Volicer L (2009). Hospice Access for Individuals With Dementia. Am J Alzheimers Dis Dementias®.

[4] Mitchell SL, Teno JM, Kiely DK, Shaffer ML, Jones RN, Prigerson HG, et al. The clinical course of advanced dementia. N Engl J Med. 2009;361(16):1529–38. 10.1056/NEJMoa0902234. 10.1056/NEJMoa0902234 PMC2778850 19828530

[5] Shega JW, Hougham GW, Stocking CB, Cox-Hayley D, Sachs GA (2003). Barriers to limiting the practice of feeding tube placement in advanced dementia. J Palliat Med.

[6] Evers MM, Purohit D, Perl D, Khan K, Marin DB (2002). Palliative and aggressive end-of-life Care for Patients with Dementia. Psychiatr Serv.

[7] Borbasi J (2016). Life before death : improving palliative care for older Australians / Dr Jessica Borbasi.

[8] Lee RP, Bamford C, Poole M, McLellan E, Exley C, Robinson L (2017). End of life care for people with dementia: the views of health professionals, social care service managers and frontline staff on key requirements for good practice. PLoS One.

[9] Kupeli N, Leavey G, Moore K, Harrington J, Lord K, King M, et al. Context, mechanisms and outcomes in end of life care for people with advanced dementia. BMC Palliat Care. 2016;15(1):31. 10.1186/s12904-016-0103-x. 10.1186/s12904-016-0103-x PMC4785626 26965309

[10] Hill E, Savundranayagam MY, Zecevic A, Kloseck M (2018). Staff Perspectives of Barriers to Access and Delivery of Palliative Care for Persons With Dementia in Long-Term Care. Am J Alzheimers Dis Dementias®.

[11] De Witt JB, Brazil K, Passmore P, Buchanan H, Maxwell D, Mcilfactrick SJ (2017). Nurses’ experiences of pain management for people with advanced dementia approaching the end of life: a qualitative study. J Clin Nurs.

[12] Birch D, Draper J (2008). A critical literature review exploring the challenges of delivering effective palliative care to older people with dementia. J Clin Nurs.

[13] Chang E, Daly J, Johnson A, Harrison K, Easterbrook S, Bidewell J, et al. Challenges for professional care of advanced dementia. Int J Nurs Pract. 2009;15(1):41–7. 10.1111/j.1440-172X.2008.01723.x. 10.1111/j.1440-172X.2008.01723.x 19187168

[14] Beck E-R, McIlfatrick S, Hasson F, Leavey G (2017). Nursing home manager’s knowledge, attitudes and beliefs about advance care planning for people with dementia in long-term care settings: a cross-sectional survey. J Clin Nurs.

[15] Brinkman-Stoppelenburg A, Rietjens JA, van der Heide A (2014). The effects of advance care planning on end-of-life care: a systematic review. Palliat Med.

[16] Collingridge Moore D, Payne S, Van den Block L, Ling J, Froggatt K, PACE (2020). Strategies for the implementation of palliative care education and organizational interventions in long-term care facilities: a scoping review. Palliat Med.

[17] Tong A, Sainsbury P, Craig J (2007). Consolidated criteria for reporting qualitative research (COREQ): a 32-item checklist for interviews and focus groups. Int J Qual Health Care.

[18] Dyer SM, Liu E, Gnanamanickam ES, Milte R, Easton T, Harrison SL, et al. Clustered domestic residential aged care in Australia: fewer hospitalisations and better quality of life. Med J Aust. 2018;208(10):433–8. 10.5694/mja17.00861. 10.5694/mja17.00861 29848247

[19] Braun V, Clarke V (2006). Using thematic analysis in psychology. Qual Res Psychol.

[20] Andrews S, McInerney F, Robinson A (2009). Realizing a palliative approach in dementia care: strategies to facilitate aged care staff engagement in evidence-based practice. Int Psychogeriatr.

[21] Forbes S, Bern-Klug M, Gessert C (2000). End-of-life decision making for nursing home residents with dementia. J Nurs Scholarsh.

[22] Sellars M, Silvester W, Masso M, Johnson CE (2015). Advance care planning in palliative care: a national survey of health professionals and service managers. Aust Health Rev.

[23] Carter G, van Der Steen JT, Galway K, Brazil K (2017). General practitioners’ perceptions of the barriers and solutions to good-quality palliative care in dementia. Dementia..

[24] Robison J, Curry L, Gruman C, Porter M, Henderson CR, Pillemer K (2007). Partners in Caregiving in a special care environment: cooperative communication between staff and families on dementia units. The Gerontologist.

[25] Kim SK, Park M (2017). Effectiveness of person-centered care on people with dementia: a systematic review and meta-analysis. Clin Interv Aging.

[26] Lee KH, Lee JY, Kim B. Person-centered Care in Persons Living with dementia: a systematic review and meta-analysis. Gerontologist. 2020;20(20):1–12. 10.1093/geront/gnaa207 PMC9019632 33326573

[27] Lawrence V, Samsi K, Murray J, Harari D, Banerjee S (2011). Dying well with dementia: qualitative examination of end-of-life care. Br J Psychiatry.

[28] Lopez RP, Amella EJ, Mitchell SL, Strumpf NE (2010). Nurses’ perspectives on feeding decisions for nursing home residents with advanced dementia. J Clin Nurs.

[29] Gjerberg E, Lillemoen L, Forde R, Pedersen R (2015). End-of-life care communications and shared decision-making in Norwegian nursing homes - experiences and perspectives of patients and relatives. BMC Geriatr.

[30] Caron C, Griffith J, Arcand M (2005). Decision making at the end of life in dementia: how family caregivers perceive their interactions with health care providers in long-term-care settings. J Appl Gerontol.

[31] Petriwskyj A, Gibson A, Parker D, Banks S, Andrews S, Robinson A (2014). A qualitative metasynthesis: family involvement in decision making for people with dementia in residential aged care. Int J Evid Based Healthc.

